# Imaging evolution of pancreatic intraductal oncocytic papillary neoplasm presenting with recurrent pancreatitis: A case report and literature review

**DOI:** 10.3892/etm.2026.13322

**Published:** 2026-10-02

**Authors:** Ying Liu, Hao Yu, Lianjun Zhou, Lin Wang

**Affiliations:** Department of CT/MRI, Ningcheng County Central Hospital, Chifeng, Inner Mongolia 024200, P.R. China

**Keywords:** intraductal oncocytic papillary neoplasm, pancreas, CT, MRI, cystic pancreatic neoplasm

## Abstract

Intraductal oncocytic papillary neoplasm (IOPN) of the pancreas is a distinct type of pancreatic intraductal epithelial neoplasm with malignant potential. Although the long-term prognosis is generally favorable, accurate preoperative diagnosis remains a challenge. The present study reported the case of a 54-year-old female patient with an initial clinical manifestation of recurrent pancreatitis. Serial contrast-enhanced CT performed over 2 consecutive years documented progressive enlargement of the pancreatic lesion from 2.5 to 3.5 cm along with parallel growth of the intralesional solid mural nodule from 1.1 to 2.1 cm, newly developed main pancreatic duct dilatation and relative hypoenhancement of the nodule compared with normal pancreatic parenchyma despite visible contrast uptake. After the patient underwent radical pancreatic resection, postoperative histopathology confirmed pancreatic IOPN with high-grade dysplasia with no evidence of invasive carcinoma. IOPN lacks pathognomonic clinical features; typical imaging findings include mixed cystic-solid lesions with mural nodules, variable pancreatic duct dilatation, the absence of peripancreatic neoplastic invasion and mural nodules demonstrating moderate enhancement inferior to surrounding pancreatic tissue. The present report systematically summarizes the clinicoradiological and pathological characteristics of pancreatic IOPN and discusses diagnostic strategies for differentiating IOPN from other pancreatic intraductal cystic neoplasms.

## Introduction

Intraductal oncocytic papillary neoplasm (IOPN) is a rare, genetically distinct intraductal epithelial neoplasm of the pancreas with malignant potential. Under previous classification systems, IOPN was grouped as a morphological variant of intraductal papillary mucinous neoplasm (IPMN); however, the 2019 World Health Organization (WHO) Classification of Digestive System Tumors reclassified IOPN as an independent diagnostic entity due to profound discrepancies in histomorphology and molecular signatures (1). Epidemiological analysis has indicated that IOPN accounts for only 1-8% of all pancreatic IPMN-spectrum lesions (1). Adsay *et al* (2) first published a series of 11 cases of pancreatic IOPN in 1996, noting clinical and imaging phenotypes that overlapped with conventional IPMN. Subsequent genomic research has identified core molecular differences between these conditions: Unlike IPMN, which frequently harbors *KRAS* and *GNAS* driver mutations, pancreatic IOPN is characterized by recurrent protein kinase cAMP-activated catalytic subunit alpha (*PRKACA*) or *PRKACB* gene fusions, with minimal mucin secretion representing a key morphological distinction (3,4).

Accumulating evidence from clinical series indicates that the long-term prognosis of IOPN is generally favorable (4-6). Nevertheless, precise preoperative radiological diagnosis is critical for risk stratification and individualized surgical planning. The present study reported a case of surgically resected pancreatic IOPN that initially manifested as recurrent pancreatitis and described its evolution on CT and MRI findings acquired over a 2-year period. The recent literature was also reviewed to improve multidisciplinary recognition of this rare pancreatic neoplasm.

## Case report

### Case presentation

A 54-year-old female patient presented to the Ningcheng County Central Hospital (Chifeng, China) in March 2024 for further diagnosis and treatment of recurrent, spontaneous, distending upper abdominal pain without shoulder-back radiation, that had been reported for the past 3 months. The patient had been previously admitted to Ningcheng County Central Hospital (Chifeng, China) multiple times for acute pancreatitis. An association between the recurrent pancreatitis and a cystic intraductal lesion was suspected, although a definite causal relationship could not be established.

The medical history of the patient did not include hypertension, coronary artery disease, type 2 diabetes, viral hepatitis, tuberculosis or other infectious disorders. Furthermore, the patient had no prior abdominal surgery, trauma, blood transfusion, known drug allergies, occupational heavy metal exposure, familial pancreatic neoplasia or hereditary digestive tract tumors. The vaccination records of the patient were unavailable.

### Physical examination

Physical examination revealed tenderness on palpation of the left upper quadrant, without rebound tenderness or muscular guarding. The liver and spleen were non-palpable below the costal margin. Abdominal percussion was tympanic, shifting dullness was absent, hepatic dullness was preserved and there was no tenderness to percussion over the liver, spleen or bilateral kidneys. Bowel sounds were normal, with no succussion splash or other signs indicating an air-fluid level. Bilateral lower limb edema or focal neurological deficits were not observed.

### Ultrasound and laboratory findings

Abdominal ultrasound revealed a mixed cystic-solid pancreatic mass, a separate cyst in the pancreatic tail, dilatation of the segmental pancreatic duct and concurrent hepatic steatosis. Serum biochemistry and tumor marker test results exhibited a mildly elevated CA19-9 level of 40.27 U/ml (reference range: 0.00-27.00 U/ml). CEA, serum amylase, lipase, liver function panel, total/direct bilirubin and systemic inflammatory markers (including CRP and white blood cell count) were also assessed during episodes of pancreatitis. Serum amylase was reported to be 1504.80 U/l (reference range: 35.00-135.00 U/l) and lipase was reported to be 2614.30 U/l (reference range: 13.00-60.00 U/l). All other laboratory parameters were within their normal limits.

### Sequential contrast-enhanced CT imaging

All CT scans were performed using a multi-detector row CT with uniform slice thickness, non-ionic intravenous contrast medium and four-phase acquisition (unenhanced, arterial, portal venous and delayed equilibrium phase). Initial unenhanced and contrast-enhanced abdominal CT was performed in March 2022, during the patient's first hospitalization for acute pancreatitis. It revealed a well-circumscribed round hypoattenuating lesion within the pancreatic body, measuring 1.7x2.0x2.5 cm. An internal solid mural nodule measuring 1.1x0.7x1.1 cm was also present. Perilesional peripancreatic inflammatory fat stranding was visible. The main pancreatic duct exhibited no dilatation at this time (Fig. 1).

Follow-up contrast-enhanced CT performed in March 2024 revealed that the primary lesion had enlarged to 2.2x2.5x3.5 cm, forming an exophytic contour inferior to the pancreatic parenchyma. The intralesional mural nodule had expanded to 1.0x1.0x2.1 cm. Segmental dilatation of the pancreatic body-tail duct was also observed, with a maximum diameter of 0.7 cm. Quantitative attenuation measurements determined that the mural nodule demonstrated intrinsic contrast enhancement across all post-contrast phases, but remained relatively hypoattenuating compared with the adjacent normal pancreatic tissue in the arterial and venous phases (Fig. 2). Communication between the cystic lesion and the dilated pancreatic duct was subsequently verified through magnetic resonance cholangiopancreatography (Fig. 3).

### MR plain and contrast-enhanced imaging (Fig. 3)

Pancreatic MRI with gadolinium-based contrast and dedicated MRCP sequences was performed, including T1-weighted imaging (T1WI), T2WI, diffusion-weighted imaging (DWI) and apparent diffusion coefficient (ADC) mapping. The pancreatic body lesion appeared as a well-demarcated multicystic mass with prolonged T1/T2 signal intensity and focal exophytic bulging. The internal mural nodules showed mild DWI hyperintensity with limited ADC diffusion restriction. Direct communication between the lesion and the segmentally dilated pancreatic body-tail duct was clearly visualized on MRCP. The enhancement patterns of the mural nodule were consistent with the CT findings. Peripancreatic inflammatory fat stranding secondary to pancreatitis was clearly distinguished from neoplastic invasion of the peripancreatic fat.

### Surgical procedure, gross pathology and histopathological diagnosis

The patient underwent distal pancreatectomy with splenectomy. Microscopic examination of the resection margins was negative for neoplastic epithelium. A number of peripancreatic tissue samples were excised intraoperatively and processed for histological examination; no dedicated lymph node dissection was performed and no metastatic carcinoma was identified in the sampled peripancreatic tissues. The resected specimen measured 9.5x3.0x2.5 cm, with a maximum lesion diameter of 3.5 cm. No gross imaging of the resected specimen was available. Light microscopy revealed complex arborizing papillary structures with central fibrovascular cores, which were lined by oncocytic cuboidal and columnar epithelial cells. These tumor cells exhibited abundant granular eosinophilic cytoplasm, enlarged round nuclei and prominent nucleoli, presenting typical architectural and cytological features of high-grade dysplasia (Fig. 4). No invasive neoplastic extension penetrating the pancreatic duct wall was identified upon comprehensive histological examination.

### Histopathological processing

The resected specimen was fixed in 10% neutral buffered formalin at room temperature for 24 h. Fixed tissues were embedded in paraffin and serial sections of 4-µm thickness were cut. The sections were dewaxed in xylene, rehydrated through a graded ethanol series and stained with Harris hematoxylin at room temperature for 5 min for nuclear counterstaining, followed by differentiation in acid alcohol, bluing in running tap water, and counterstaining with eosin Y at room temperature for 1-2 min. Stained sections were dehydrated through graded ethanols, cleared in xylene and mounted in neutral balsam (7).

The final standardized pathological diagnosis was pancreatic IOPN with high-grade dysplasia, without associated invasive adenocarcinoma. Immunohistochemical staining and molecular testing were not performed in the present case. The diagnosis of IOPN was rendered according to the original pathology report and established on the basis of characteristic histological morphology on H&E staining, including complex arborizing papillae with fibrovascular cores lined by oncocytic epithelial cells with abundant granular eosinophilic cytoplasm, enlarged round nuclei and prominent nucleoli, consistent with the diagnostic criteria established by the 2019 WHO Classification of Digestive System Tumors (3). The absence of invasive carcinoma was determined via thorough sampling of the lesion.

### Postoperative follow-up

Regular surveillance, including serum CA19-9 measurement, abdominal CT and MRCP, was scheduled every 6 months postoperatively. No surgical complications, local recurrence or distant metastasis occurred during the follow-up period. To date, 42 months have elapsed since surgery and the patient remains disease-free without surgical-associated complications, local recurrence or distant metastasis.

## Discussion

IOPN is a rare intraductal pancreatic neoplasm characterized by oncocytic epithelium that forms papillary architectures. More than 200 cases of pancreatic and biliary IOPN have been reported in the literature to date, with no significant sex predilection. The reported age at onset ranges from 36 to 84 years, with a median age of 61 years (6). The clinical manifestations of IOPN overlap substantially with conventional IPMN, including recurrent pancreatitis, non-specific abdominal pain, palpable abdominal mass and complete absence of clinical symptoms (8). In the present case, recurrent pancreatitis served as the initial presentation. At present, serum tumor markers lack diagnostic specificity for IOPN. Mild elevation of CA19-9 and CEA have been reported in a minority of patients (9), consistent with the mildly elevated CA19-9 level in the patient of the present study.

IOPN may arise in any segment of the pancreas; the pancreatic head is the most common site (67% of cases), followed by the pancreatic body and tail. Approximately 5% of lesions involve the entire pancreas. IOPN may be classified as main duct-type, branch duct-type or mixed-type, analogous to IPMN classification systems (10). The hallmark imaging morphology of IOPN is mixed cystic-solid masses with internal mural nodules, which are attributed to low mucin secretion that limits diffuse pancreatic duct dilatation. Reported maximum lesion diameters range from 1 to 15 cm, with a mean size of 5.5 cm at diagnosis (6).

In the present case, serial imaging over a 2-year period captured stepwise disease progression: An initially small cystic-solid lesion with an isolated mural nodule without duct dilatation, followed by lesion and nodule enlargement and secondary pancreatic duct dilatation associated with high-grade dysplasia. Prior research has confirmed mural nodules ≥5 mm in maximal diameter as high-risk imaging signs associated with underlying high-grade dysplasia or invasive carcinoma (11). However, it must be emphasized that enhancing mural nodules are not pathognomonic for IOPN, as this feature may occur in various cystic pancreatic neoplasms (4).

Regarding differential diagnosis, the following may be considered: i) IPMN/mucinous intraductal papillary carcinoma (IPMC): Clinical symptoms overlap broadly with those of IOPN, although elevated CA19-9 and CEA levels are more pronounced in malignant IPMC (12). Imaging typically shows diffuse pancreatic duct dilatation secondary to abundant mucin production, with smaller cystic locules and higher rates of peripancreatic invasion, postoperative recurrence and inferior overall survival compared with IOPN. International consensus guidelines (1) stratify IPMN risk based on mural nodule size and duct caliber. ii) Intraductal tubulopapillary neoplasm (ITPN): Similar to IOPN, ITPN produces minimal mucin secretion. However, ITPN is defined by tubular rather than papillary epithelial proliferation. The imaging hallmarks of ITPN include the two-tone duct sign and the wine-cork filling defect sign on MRCP (13,14). iii) Other entities in the differential diagnosis include mucinous cystic neoplasm, serous cystic neoplasm, solid pseudopapillary neoplasm and cystic pancreatic neuroendocrine neoplasm (5,11,12). Accurate diagnosis requires comprehensive integration of the lesion location, duct communication status, intralesional mucin volume, internal architecture, mural nodule enhancement kinetics, cytological sampling, histomorphology, and immunophenotype and molecular profiles, rather than relying solely on duct dilatation or mural nodule presence.

IOPN is recognized to have malignant potential, with 29% of resected cases complicated by concurrent invasive adenocarcinoma. Surgical resection is indicated for all radiologically resectable IOPN lesions with high-risk imaging features. Operative planning must be individualized based on the tumor location, lesion extension and patient functional status. Pancreaticoduodenectomy (Whipple procedure) is typically performed for pancreatic head IOPN, whereas distal pancreatectomy (with or without splenectomy) is performed for body/tail lesions. Routine adjuvant radiotherapy is not universally recommended for cases of IOPN-associated invasive carcinoma. Decisions regarding adjuvant management for patients with invasive disease should be individualized and determined via multidisciplinary tumor board discussion (5). Prognosis differs significantly between non-invasive IOPN and IOPN with invasive adenocarcinoma. Non-invasive IOPN is associated with excellent long-term survival. A previous study (15) reported a 100% 5-year overall survival rate and a 66% 5-year disease-free survival rate for invasive IOPN, with 46% local recurrence within 10 years post-resection. Repeat salvage surgical resection may be required to maintain favorable long-term outcomes (15). Although the overall prognosis remains favorable, invasive IOPN exhibits distinct biological features that cannot be uniformly summarized as indolent without stratification (5).

Several limitations of the present case report should be acknowledged. First, immunohistochemical staining was not performed, which precludes formal confirmation of oncocytic and pancreatobiliary differentiation and limits direct immunophenotypic comparison with previously reported IOPN series (4,14,15). Although the histological morphology was sufficiently characteristic to establish the diagnosis on H&E staining alone, the addition of immunohistochemical markers (such as hepatocyte paraffin 1, CD117, mucin 1 and cytokeratin 7) would have strengthened diagnostic confidence, particularly in distinguishing IOPN from its mimics. Second, molecular testing for *PRKACA* or *PRKACB* gene fusions was not performed; these rearrangements are highly characteristic of IOPN and could have provided molecular confirmation. Third, the postoperative follow-up duration was relatively limited and longer surveillance would be needed to fully assess long-term recurrence risk. These limitations should be considered when interpreting the findings and comparing them with the published literature.

In conclusion, IOPN is a distinct pancreatic intraductal epithelial neoplasm with malignant potential but a generally favorable long-term prognosis. Nevertheless, preoperative radiological diagnosis remains challenging. The patient of the present study initially presented with recurrent pancreatitis. Serial imaging over 2 years documented progressive enlargement of a mixed cystic-solid lesion and an internal mural nodule, new-onset pancreatic duct dilatation, persistent absence of peripancreatic neoplastic invasion and mural nodules showing intrinsic contrast enhancement with relative hypoattenuation/hypointensity compared with normal pancreatic parenchyma on CT and MRI. Multidisciplinary integration of clinical history, standardized cross-sectional imaging, endoscopic ultrasound cytology, histopathology and molecular testing is necessary for accurate preoperative differentiation from other pancreatic cystic neoplasms and to inform individualized surgical management.

## Figures and Tables

**Figure 1:**
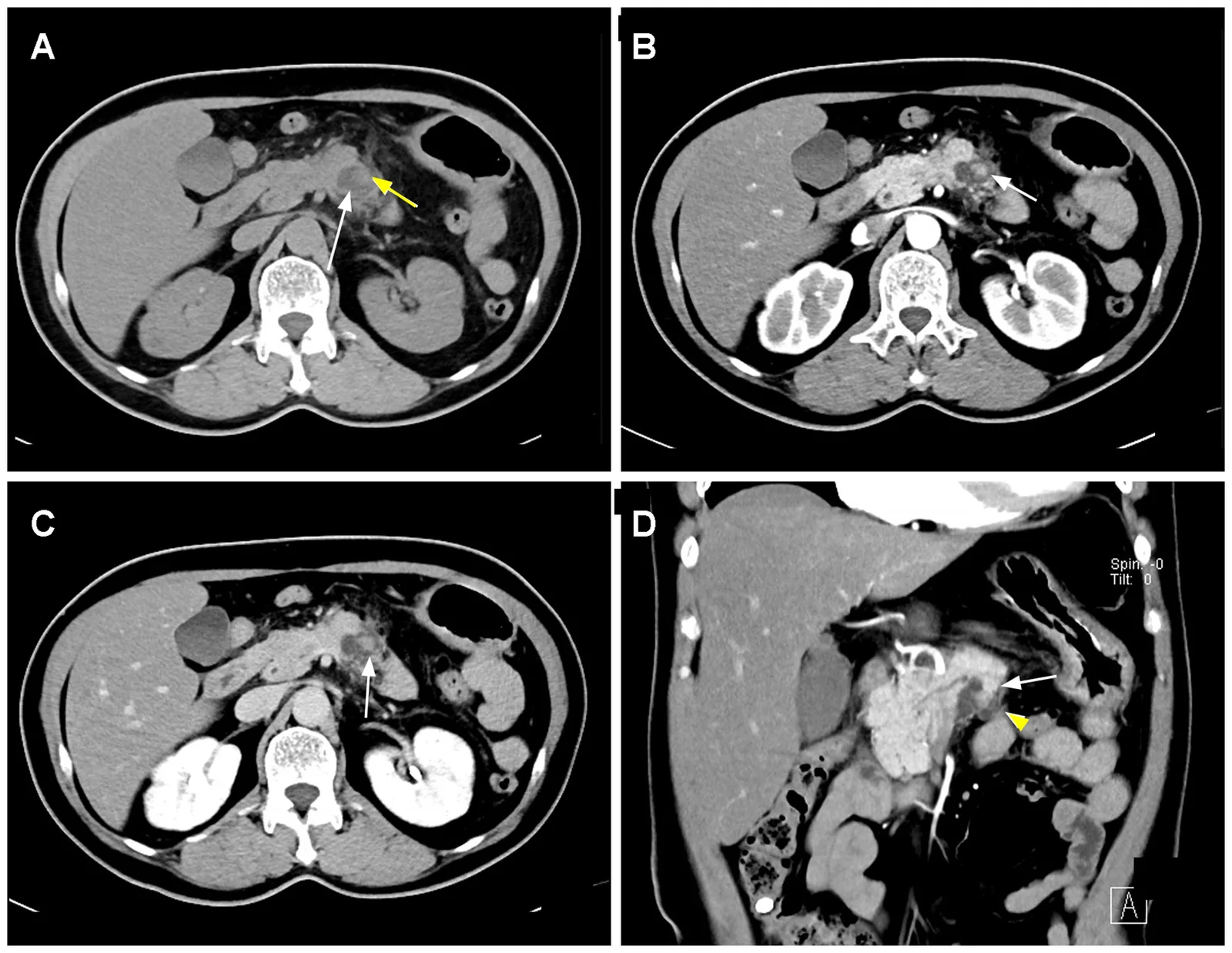
Contrast-enhanced CT of the pancreas obtained in March 2022, during the patient's first admission for acute pancreatitis (~2 years before the present case). (A) Axial unenhanced CT image showing a well-circumscribed round hypoattenuating cystic lesion (2.5 cm in maximal diameter) in the pancreatic body (white arrow), containing a small internal soft-tissue mural nodule (arrowheads). (B) Axial arterial-phase CT image. The mural nodule (white arrow) demonstrates contrast enhancement but remains relatively hypoattenuating compared with the surrounding normal pancreatic parenchyma. (C) Axial delayed-phase CT image. The cystic lesion and mural nodule (white arrow) are shown; no pancreatic duct dilatation is identified. (D) Coronal reformatted arterial-phase CT image. The lesion (white arrow) is located in the pancreatic body, with peripancreatic inflammatory fat stranding (yellow arrow) consistent with concurrent acute pancreatitis. The main pancreatic duct was not dilated at this time point.

**Figure 2:**
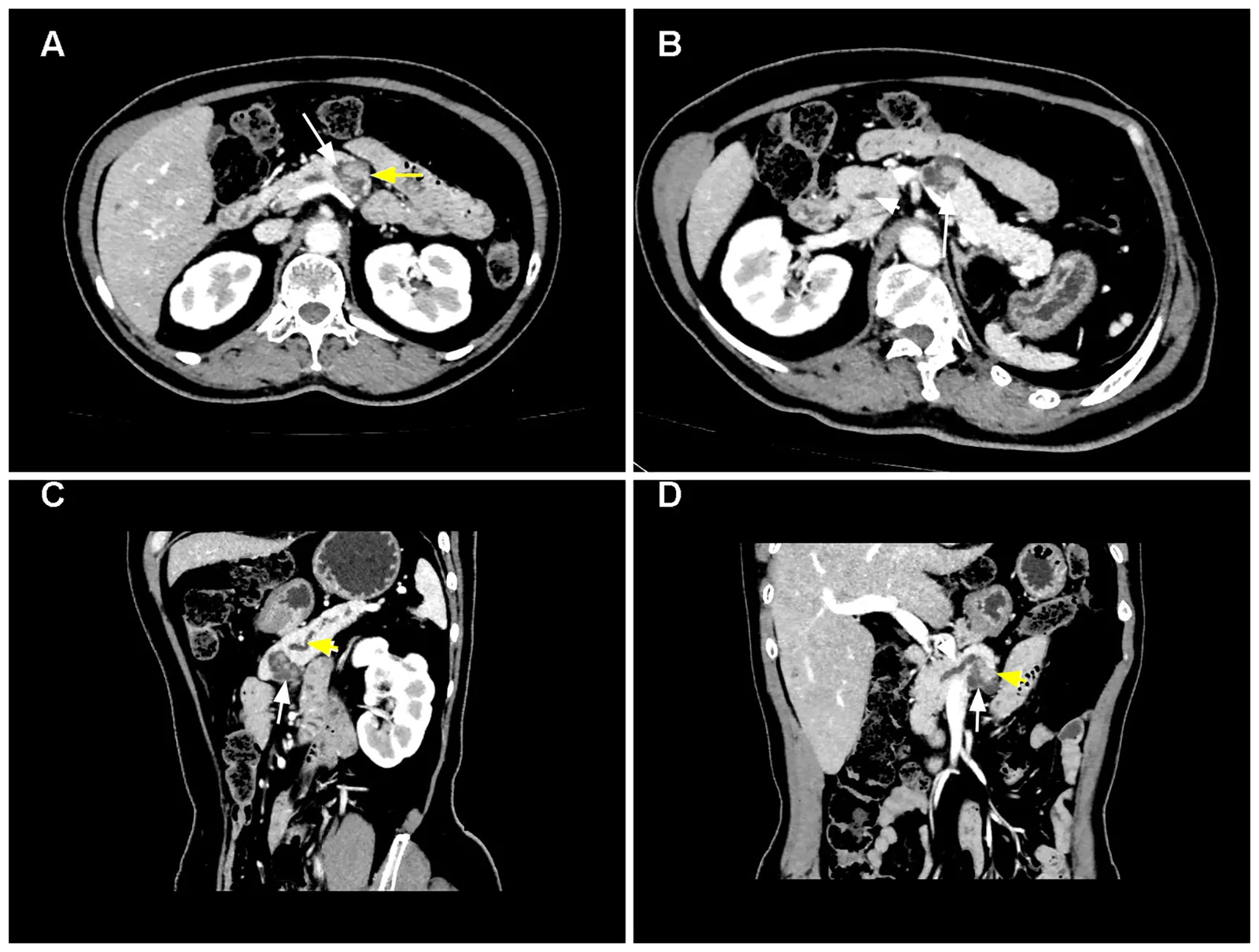
Follow-up contrast-enhanced CT of the pancreas obtained in March 2024, demonstrating interval growth of the pancreatic body lesion. (A) Axial arterial-phase CT image. The cystic lesion (white arrow) has enlarged to 3.5 cm and the internal mural nodule (yellow arrow) has increased to 2.1 cm; the nodule enhances but remains hypoattenuating relative to the surrounding pancreatic parenchyma. (B) Axial reformatted arterial-phase CT image. The mural nodules (white arrows) are shown. (C) Oblique sagittal reformatted arterial-phase CT image. The lesion (white arrow) is seen in the pancreatic body with an exophytic contour; the dilated pancreatic duct (yellow arrow) is visible proximally and distally to the lesion. (D) Coronal reformatted portal venous-phase CT image. The cystic-solid lesion (white arrow) in the pancreatic body with the enlarged mural nodule (yellow arrow) and the dilated pancreatic body-tail duct (white arrowheads; maximal caliber of 0.7 cm) are demonstrated.

**Figure 3:**
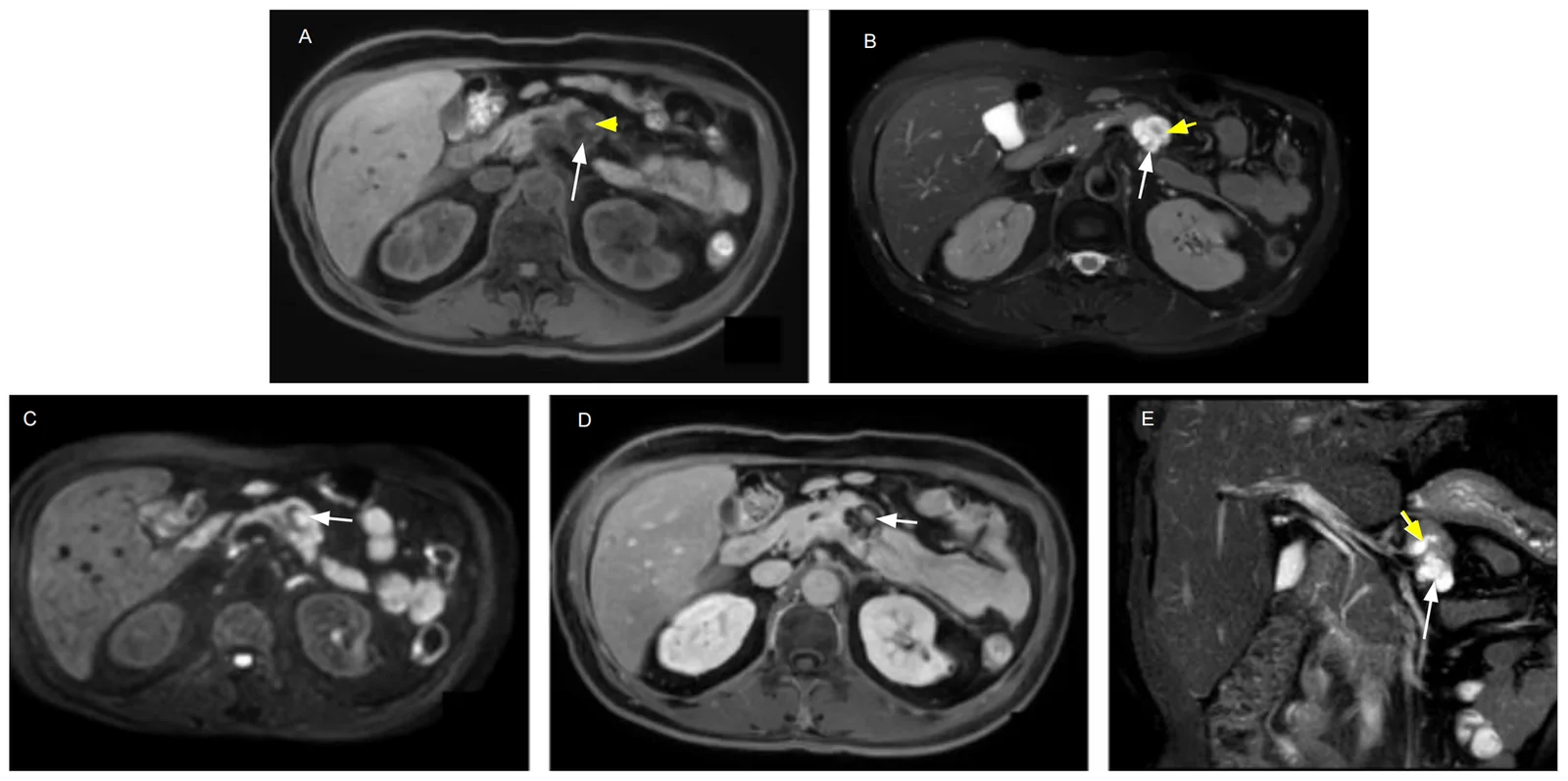
Contrast-enhanced MRI of the pancreas obtained in March 2024, demonstrating the pancreatic body lesion and its communication with the dilated pancreatic duct. (A) Axial T1-weighted image. The multicystic lesion in the pancreatic body (white arrow) is hypointense, with an internal mural nodule (yellow arrow) showing signal intensity isointense to the pancreatic parenchyma. (B) Axial T2-weighted image. The cystic lesion (white arrow) is markedly hyperintense with well-defined margins; the internal mural nodule (yellow arrow) is slightly hyperintense. (C) Axial diffusion-weighted image (b=800 sec/mm^2^; left) with corresponding ADC map (right). The mural nodule (white arrow) shows mild hyperintensity on DWI with mildly reduced signal on the ADC map, indicating limited true diffusion restriction. (D) Axial gadolinium-enhanced delayed-phase image. The mural nodule (white arrow) demonstrates contrast enhancement but remains hypointense relative to the surrounding pancreatic parenchyma. (E) Coronal T2-weighted image (magnetic resonance cholangiopancreatography-like view). The cystic lesion (white arrow) clearly communicates with the segmentally dilated main pancreatic duct (yellow arrow), demonstrating an intraductal location of the lesion. ADC, apparent diffusion coefficient.

**Figure 4:**
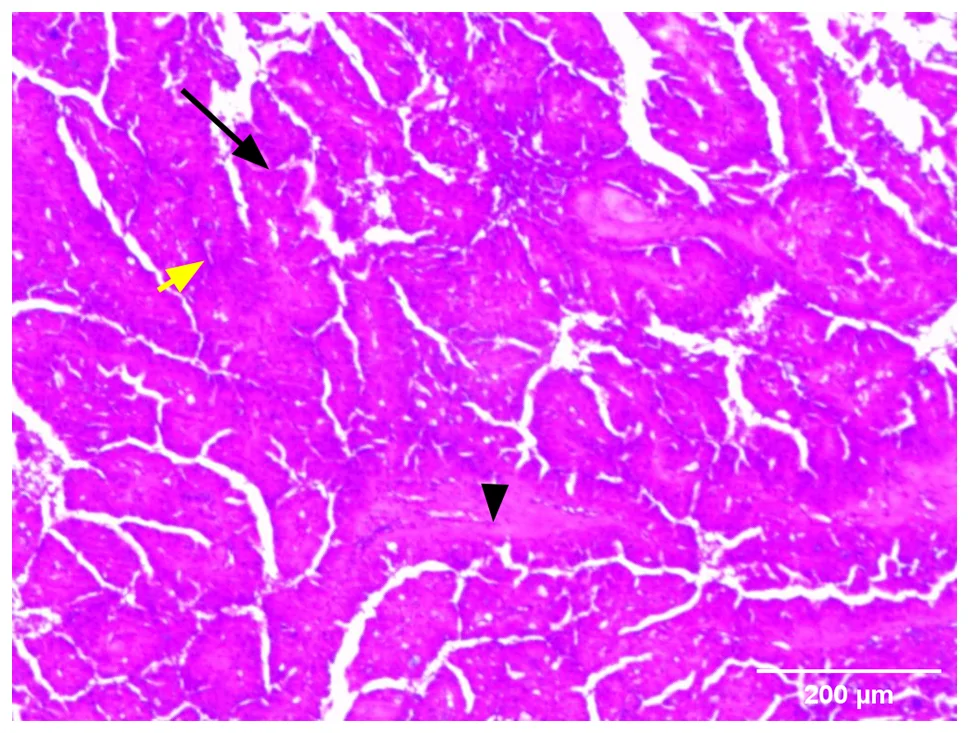
Histopathology of the resected lesion (H&E staining; original magnification, x40). The dilated pancreatic duct shows complex arborizing papillae (long black arrow) with fibrovascular cores (black arrowheads), lined by oncocytic cells with abundant granular eosinophilic cytoplasm, enlarged nuclei and prominent nucleoli (yellow arrow), consistent with high-grade dysplasia. No invasion through the duct wall was identified.

## Data Availability

The data generated in the present study are included in the figures and/or tables of this article.
